# Video-assisted thoracoscopic surgery is more favorable than thoracotomy for administration of adjuvant chemotherapy after lobectomy for non-small cell lung cancer

**DOI:** 10.1186/1477-7819-9-170

**Published:** 2011-12-21

**Authors:** Guanchao Jiang, Fan Yang, Xiao Li, Jun Liu, Jianfeng Li, Hui Zhao, Yun Li, Jun Wang

**Affiliations:** 1 Department of Thoracic Surgery, Center for Mini-invasive Thoracic Surgery, People's Hospital, Peking University, #11 Xizhimen South Street, Beijing, China, 100044

**Keywords:** video-assisted thoracoscopic surgery (VATS), lobectomy, non-small cell lung cancer (NSCLC), adjuvant chemotherapy

## Abstract

**Background:**

Video-assisted thoracoscopic surgery (VATS) lobectomy is a newly developed type of surgery for lung cancer and has been demonstrated obvious minimally-invasive advantages compared with traditional thoracotomy. Theoretically, that less trauma leads to quicker recovery and may facilitate administration of adjuvant chemotherapy. We tested this hypothesis in this study.

**Methods:**

One hundred and ten NSCLC patients underwent lobectomy and adjuvant chemotherapy from June 2004 to June 2010 was analyzed. The baseline characteristic criteria, variables related to surgery and accomplishing status of chemotherapy were analyzed.

**Results:**

All 110 patients underwent lobectomy through VATS (n = 54) or thracotomy (n = 56) and adjuvant chemotherapy. There was no significant difference in patients' age, preoperative pulmonary function, co-morbidity, pathologic staging between the two groups, whereas, blood loss, operation time and postoperative complications, chest tube duration and length of stay were less in VATS group. There were no significant differences in time to initiation chemotherapy. Cases in VATS group received more cycles of chemotherapy (3.6 vs. 3.0, p = 0.002). A higher proportion of patients received full dose on schedule in VATS group (57.4% vs. 33.9%, p = 0.013) and a higher proportion of patients completed ≥75% planed dose, (88.9% vs. 71.4%, p = 0.022); slightly higher proportion of patients in thoracotomy group had grade 3 or more toxicity (20.4% vs. 35.7%, p = 0.074).

**Conclusions:**

Patients underwent lobectomy by VATS have better compliance and fewer delayed or reduced dose on adjuvant chemotherapy than those by thoracotomy.

## Background

Adjuvant cisplatin-based chemotherapy is recommended for patients with stages II, IIIA, and a subgroup of IB non-small cell lung cancer (NSCLC), based on the positive results of several large randomized trials and a meta-analysis [1-4]. Among these studies, the JBR10 study reported that postoperative chemotherapy increased 15 percent at 5 years survival rate in lung cancer patients (mainly for patients with stage II disease) [2]; ANITA study reported that the absolute overall survival benefit with adjuvant chemotherapy was 8.6 percent at five years and 8.4 percent at seven years (mainly for patients with stage II and IIIA disease) [3]. Subgroup analysis of CALGB 9633 indicated that lung cancer patients in stage IB with tumor diameter more than 4 cm may benefit from postoperative chemotherapy [4].

Although no direct evidence, it is postulated that early and full-dose adjuvant chemotherapy may maximize the benefit. However, patients receiving lobectomy through conventional thoracotomy sometimes have delayed adjuvant therapy due to slow recovery, and not infrequently, discontinue treatment because of worsening of performance status (PS) [4]. During the past decade, more and more evidence showed that video-assisted thoracoscopic surgery (VATS) lobectomy, also referred to as thoracoscopic lobectomy, can be an alternative surgical approach for lung cancer patients [5-12]. The advantages of VATS lobectomy include remission of post-operative pain, less releasing of inflammatory factor and less effect on pulmonary functions, fewer complications and rapid recovery [6-10], without compromising the long term survival and disease free survival [11,12]. However further studies are needed to evaluate the minimally-invasiveness of VATS on adjuvant chemotherapy delivery.

In this study, we retrospectively analyzed 110 NSCLC patients who received thoracotomy or VATS lobectomy since June 2004 in our hospital. We compared their accomplishing status of postoperative chemotherapy to investigate whether the postoperative chemotherapy compliance is better in patients underwent VATS than those through thoracotomy.

## Materials and methods

### 1. Patient Selection

The medical record of 497 NSCLC patients who underwent lobectomy by VATS (n = 243) or by thoracotomy (n = 254) from June 2004 to June 2010 in Peking University People's Hospital were reviewed. The indications for VATS lobectomy were as follows: clinical stage I or II peripheral non-small cell lung cancer without involvement of chest wall; no calcified hilar lymph nodes on CT scan; tumor less than 5 cm in diameter. To make the thoracotomy group comparable, only patients meet the same preoperative criteria were selected. Patients underwent lobectomy and systemic mediastinal lymph nodes dissection, and at least one cycle of adjuvant chemotherapy in our institution were elligible for this the current study, patients received neoadjuvant therapy, bi-lobectomy or pneumonectomy were excluded. As intention to treat principle, conversion-to-thoracotomy cases were analyzed in the VATS group. Finally, 110 patients were enrolled, of them 54 patients underwent VATS, the other 56 patients, thoracotomy. There were 2 conversion-to-thoracotomy cases in VATS group, of them, one case was due to inflammation induced peri-vascular fibrosis and interlobar lymph node calcification; the other conversion was caused by intra-operative bleeding. This study was conducted with Institutional Review Board approval. Consent from patients was waived because patients were not identified individually.

### 2. Surgical Technique

VATS lobectomy was performed in 54 patients in this group (including two cases of conversion to thoracotomy). We used three-port technique for anatomic lobectomy without rib-spreading, the utility incision was usually 4-5 cm in length in order to retrieve the specimen [13]. Systemic lymph nodes dissection was also carried out with the standard identical to thoracotomy surgery. Open lobectomy was performed on 56 patients with a 15-20 cm lateral incision in the 4th or 5th intercostal space without rib transection, but partial spreading of the serratus and latissimus muscles. Thoracotomy incision was closed with running pericostal suture as the first layer, while VATS incisions, the muscular.

Pathologic staging was performed according to the 6th International Staging System for Lung Cancer. Patients were followed from the date of the operation until either death or April 2011. Survival was calculated, and adverse events, including all causes of death, were evaluated.

### 3. Chemotherapy administered

Adjuvant chemotherapy was recommended to patients with pathologic stage Ib or higher disease after recovery from surgery and ECOG performance status (PS) 0-1. The chemotherapy regimens included gemcitabine (1250 mg/m^2^, d1, d8,) plus cisplatin (75 mg/m^2^, d1) every 3 weeks, or paclitaxel (175 mg/m^2 ^over 3 hours) plus carboplatin (area under curve [AUC] 5) every 3 weeks, and it was chosen by the treating physician. The maximum number of cycles was 4. Dose reduction or dose delay were decided according to objective criteria (white blood cell count, absolute neutrophil count and serum creatinine, side effect on gastrointestinal, liver and neurologic systems) and subjective criteria (PS score). Usually, the treatment would be delayed or dose reduced for grade 3 or higher toxicity and PS > = 2. The toxicity was graded according to the NCI Common Toxicity Criteria for Adverse Events (version 2.0).

### 4. Examination of compliance

Baseline characteristics including age, gender, ECOG performance status, co-morbidities, pulmonary function, and operative parameters such as operation time, blood loss, chest tube duration, length of stay and post operative complications, pathologic staging were compared between the two groups. Outcomes were measured with number of cycles of chemotherapy, mean dose administered to each patient; time to initiate adjuvant chemotherapy, proportion of patients received delayed or reduced doses and treatment-related toxicity.

### 5. Statistical methods

Variables were compared between the VATS and the thoracotomy groups, using Student *t *test for continuous variables, and either chi-square or Fisher's exact test for categorical variables. Survival rates were calculated by life-table analysis. Kaplan-Meier survival curves were compared using the log-rank test for univariate analysis. A possibility value of 0.05 or less was considered statistically significant. All statistical analyses were performed using SPSS 11.5 software.

## Results

### 1. Patient characteristics

The baseline characteristics of the patients are shown in Table 1. There was no significant difference in age, gender, ECOG performance status, pulmonary function and co-morbidities between the two groups. In VATS group, there were more adenocarcinoma patients (p < 0.001). With respect to perioperative parameters, the VATS group had less operation time and blood loss (p < 0.001), as well as chest tube duration (p = 0.025) and length of stay (p = 0.043). The incidence rate of postoperative complications was also slightly lower in VATS group than that in thoracotomy group, especially atrial fibrillation (Table 2).

**Table 1:** Comparison of general states between two groups of patients before chemotherapy

	VATS n = 54	Thoracotomy n = 56	*p *value
Gender (male), no. (%)	33(61.1)	38(67.9)	0.460
Age (year)	62.9 ± 9.6	61.9 ± 11.1	0.596
ECOG Performance Status, no. (%)			0.862
0	29(53.7)	31(55.4)	
1	25(46.3)	25(44.6)	
Preoperative complications, no. (%)			
Hypertension	20(37.0)	13(23.2)	0.114
Diabetes mellitus	4(7.4)	2(3.6)	0.434
Coronary heart disease	6(11.1)	3(5.4)	0.316
Disease of respiratory system	6 (11.1)	11(19.6)	0.216
Preoperative pulmonary function (percent of predicted value)			
FVC	100.8 ± 14.1	99.7 ± 19.0	0.749
FEV1	97.1 ± 15.3	97.1 ± 24.1	0.987
DLCO	85.8 ± 16.3	90.4 ± 19.5	0.174

Note: Diseases of respiratory system include old pulmonary tuberculosis, pulmonary interstitial disease and COPD

**Table 2:** Pathological types, postoperative pathologic staging and postoperative complications of the two groups of patients

	VATS n = 54	Thoracotomy n = 56	*p *value
Operation time (min)	192.0 ± 45.0	238.0 ± 63.7	<0.001*
Blood loss (ml)	225.4 ± 165.1	425.4 ± 261.3	<0.001*
No. of lymph node dissection	14.3 ± 8.8	13.0 ± 5.5	0.245
Chest tube duration (days)	8.0 ± 2.8	9.6 ± 4.4	0.025*
Postoperative length of stay (days)	10.8 ± 3.7	12.5 ± 4.8	0.043*
Complications, no. (%)			
Pneumonia	2(3.7)	5 (8.9)	0.438
Atrial fibrillation	1 (1.9)	7 (12.5)	0.061
Prolonged air leak >7days	0 (0.0)	4 (7.1)	0.118
Others	3 (5.6)	6 (10.7)	0.490
Tumor size (cm)	3.3 ± 1.4	3.8 ± 1.6	0.071
Pathological types, no. (%)			<0.001*
Adenocarcinoma	44(81.5)	24(42.9)	
Squamous cell carcinoma	8(14.8)	31(55.4)	
Others	2(3.7)	1(1.8)	
Pathologic staging, no. (%)			0.087
Ib	26 (48.1)	21 (37.5)	
II	7(13.0)	17 (30.4)	
III	21(38.9)	18 (32.1)	

### 2. Chemotherapy compliances

The chemotherapy regimens administered in two groups were similar (p = 0.577). But patients in VATS group completed more cycles of chemotherapy (3.6 vs. 3.0, p = 0.002). There were more patients completed all four cycles at full planned dose on schedule in VATS group (57.4% vs. 33.9%, p = 0.013) and a higher proportion of patients completed ≥75% planed dose, (88.9% vs. 71.4%, p = 0.022). The mean dose administered in VATS group was more than that in thoracotomy group. Reasons for dose delay or dose reduction in our series were toxicity and patients' refusal. Patients in VATS group were less likely to experience grade 3 or 4 toxicities than those in thoracotomy group (20.4% vs. 35.7%, p = 0.074) (Table 3).

**Table 3:** Chemotherapy compliance after lobectomy by different approach

	VATS n = 54	Thoracotomy n = 56	*p *value
Chemotherapy regimens, no. (%)			0.577
Gemcitabine+Cisplatin	25 (46.3)	29 (51.8)	
Paclitaxol+Carboplatin	22(40.7)	23(41.1)	
Vinorelbine+Cisplatin	7(13.0)	4(7.1)	
Time to initiate chemotherapy(days)	33.7 ± 10.9	34.0 ± 13.3	0.904
Cycles completed, (Mean ± SD)	3.6 ± 0.72	3.0 ± 1.1	0.002*
Pts. Receive full dose on schedule, no. (%)	31 (57.4)	19 (33.9)	0.013
Pts. Receive ≥75% planed dose, no. (%)	48(88.9)	40(71.4)	0.022*
Mean doses, (% of planed)			
Cisplatin (mg/m2)	267 (89.0)	237 (79.0)	0.121
Gemcitabine (mg/m2)	8900 (9.0)	7930 (79.3)	0.120
Paclitaxol (mg/m2)	620 (88.6)	495 (70.7)	0.020*
Carboplatin (AUC mg/ml/min)	21.3 (88.8)	17.0 (70.8)	0.019*
Toxicity, no. (%)			0.011*
Grade 1/2 toxicity	25 (46.3)	34 (60.7)	0.130
Grade 3/4 toxicity	11 (20.4)	20 (35.7)	0.074

All the Grade 3/4 toxicities of chemotherapy are listed in Table 4 according to NCI-CTC grade. The VATS group was less likely to suffer from hematologic (16.7% vs. 33.3%, p = 0.046) and non-hematologic toxicities (11.1% vs. 26.8%, p = 0.037) (Table 4).

**Table 4:** Grade 3/4 toxicity of chemotherapy according to NCI-CTC grade

	VATS n = 54	Thoracotomy n = 56	*p *value
Hematologic,no. (%)	9(16.7)	18(33.3)	0.046*
Leukopenia	5 (9.3)	9 (16.1)	0.284
Neutropenia	7(13.0)	14 (25.0)	0.108
Anemia	1(1.9)	0(0.0)	0.491
Thrombocytopenia	1(1.9)	0(0.0)	0.491
Non-hematologic, no. (%)	6 (11.1)	15(26.8)	0.037*
Nausea	6 (11.1)	14 (25.0)	0.059
Vomiting	3 (5.6)	5(8.9)	0.716
Fatigue/asthenia	1 (1.9)	3 (5.4)	0.618
Diarrhea/constipation	1 (1.9)	2 (3.6)	1.000
Neuropathy: sensory	1 (1.9)	1 (1.8)	1.000
Phlebitis	0 (0.0)	0 (0.0)	1.000
Infection	0 (0.0)	0 (0.0)	1.000

Although the pathologic type was different in the two groups, no significant differences of the compliance of the adjuvant chemotherapy were noted between adenocarcinoma and squamous cell carcinoma or other histology (Table 5).

**Table 5:** Adjuvant chemotherapy compliance after lobectomy stratified by pathological types

	Adeno n = 68	Squamous cell and other n = 42	*p *value
Cycles completed, (Mean ± SD)	3.3 ± 0.91	3.2 ± 1.1	0.830
Pts. Receive full dose on schedule, no. (%)	31 (45.6)	19 (54.4)	0.971
Pts. Receive ≥75% planed dose, no. (%)	55 (80.9)	33(78.6)	0.768
Toxicity, no. (%)			
Grade 1/2 toxicity	34 (50.0)	25 (59.5)	0.330
Grade 3/4 toxicity	19 (27.9)	12 (28.6)	0.943

### 3. Follow-up

Follow-ups were completed for 101 patients (101/110). Patients were followed up until death. The median follow-up period was 30 months (2-82 months). Overall 2-year survival rates were 91.0% and 70.6%, and disease-free 2-year survival rates were 70.9% and 62.7% for the VATS and thoracotomy groups, respectively. There was a trend toward better overall and disease-free survival in the VATS group, but the difference did not reach statistical significance.

## Discussion

After the publication of survival results of several adjuvant chemotherapy trials, adjuvant chemotherapy has become an important part of multimodality treatment for non-small cell lung cancer [1-4]. It is postulated that better compliance of chemotherapy may increase its efficacy and therefore would bring survival benefits [14]. If so, better compliance of adjuvant chemotherapy would be of therapeutic value. VATS lobectomy is a newly developed approach for lung cancer in the past two decades and has shown significant minimally-invasive advantages over traditional thoracotomy, mainly manifesting fewer traumas to the chest wall, remission of postoperative pain and rapid recovery [5-10]. It is unknown whether the minimally-invasive natures of the VATS can translate into better compliance of adjuvant chemotherapy. At the time we finished this manuscript, there was just one publication of a retrospective small study with similar design by Petersen et al, comparing the adjuvant chemotherapy state of 43 patients received lobectomy through thoracotomy and 57 patients underwent VATS. Their preliminary results indicated that the patients receiving VATS have better compliance of adjuvant chemotherapy than those receiving thoracotomy for lobectomy [15].

Cycles of chemotherapy completed, mean dose administered and toxicity are the key criteria to evaluate the compliance of chemotherapy. In our study, 73 patients (68.2%) completed four cycles of chemotherapy, among them overall 50 patients (45.5%) completed it at full planned dose on schedule. These numbers was comparable to those in the published large trials [2,4,16]. When comparing the two groups of different surgical approaches, the average number of cycles was higher in VATS group, also more patients in VATS group complete ≥75% planed dose. This was consistent with the findings that mean dose administered was also higher in VATS group and more patients finished four cycles chemotherapy on schedule at full dose in VATS group than in thoracotomy group. Although higher percentage of patients after VATS lobectomy received full dose chemotherapy on schedule was reported [17], this difference could be due to different patient population, the difference in management of adverse effects, and the preference of patients and physicians toward chemotherapy's efficacy and adverse effects.

The better compliance of the chemotherapy in VATS group was also represented by less severe toxicity in VATS group. It has been reported that patients after pneumonectomies were more likely to experience grade 3 or 4 toxicities than after lobectomies [16]. This means that extend of resection, or more specifically, the invasiveness of the procedure, does affect the tolerance of adjuvant therapy. Surgical trauma may just increase the likelihood of toxicity accumulation during adjuvant chemotherapy, and cause severe adverse effects, performance status worsening, and chemotherapy delay and/or dose reduction, even discontinuation, as a consequence. However, because of limited sample size, this hypothesized difference failed to reach statistical significance in the current study.

Patients receiving VATS have less surgical trauma and quicker recover; theoretically, these patients may start postoperative chemotherapy earlier. However, the results of this study indicated that patients in both groups started postoperative chemotherapy about 30 days after surgery. One reason is that there is no difference in length of stay between the two groups. Another reason is that we commonly recommend patients start chemotherapy one month after operation. This time interval is determined from experience of thoracotomy patients. In some studies, adjuvant chemotherapy usually initiated 28-50 days postoperatively [4,16]. VATS patients have quicker recovery and may start adjuvant chemotherapy earlier. More studies are needed to address this issue, especially the therapeutic benefit of early-starting adjuvant chemotherapy.

In an exploratory analysis of JBR10 study, factors affect compliance included surgery extent, gender and age [16]. Since these factors, as well as ECOG performance status, co-morbidity, pulmonary functions, postoperative complications and chemotherapy regimens were comparable between the two groups in this study, the difference of compliance for postoperative chemotherapy can be attributed to the surgical approaches, namely, better compliance after VATS than thoracotomy lobectomy.

Improved survival is the ultimate goal of adjuvant chemotherapy with better compliance. We next tried to determine if this better compliance of adjuvant chemotherapy can translate into better survival. The two groups have comparable p-stage distribution. However, the better survival in VATS group could be at least partly due to more adenocarcinomas. It is elucidated recently that about half of adenocarcinomas in eastern Asian patients carry EGFR (epidermal growth factor receptor) activating mutation, which renders better prognosis [18,19]. Another bias is more stage Ib patients in the VATS group, while more stage II of the thoracotomy patients. To make discussion about survival more complicated, if the minimal invasiveness of VATS lobectomy carries survival benefits for lung cancer is still controversial due to lacking of randomized trials. Taken together, no conclusions can be drawn from survival analysis.

We acknowledge some limitations of this study: retrospective in nature, limited sample size, and patients in the two groups were not perfectly matched.

## Conclusions

The compliance to postoperative chemotherapy is better in patients of VATS group than that in thoracotomy group. Multicenter prospective study is needed to evaluate in further whether VATS lobectomy has survival benefit through facilitating adjuvant chemotherapy.

## Competing interests

The authors declare that they have no competing interests.

## Authors' contributions

GJ and JW contributed equally to this work, both of them designed the study and wrote the whole article. FY and XL participated in the design of the study and carried out the statistics. JL and JFL participated in writing manuscript and prepared for the references. HZ and YL prepared for the collected patients' data and analyzing the data. All authors read and approved the final manuscript.
